# American Dental Association

**Published:** 1890-09

**Authors:** 


### Editorial, Etc.

WE are greatly indebted to the "*Dental Cosmos*" for the advanced proof sheets of the proceedings of the National Association of Dental Faculties, and National Association of Dental Examiners, which appeard in the last issue of the JOURNAL.

---

AMERICAN DENTAL ASSOCIATION.—The Thirtieth Annual Meeting of this Association was held at Excelsior Springs, Missouri, beginning August 5, 1890. President: M. W. Foster, of Baltimore; Secretary; George H. Cushing, of Chicago.

After the call of the roll and the adoption of a resolution dispensing with the reading of the minutes Dr. Gardiner, of Chicago, precipitated a discussion by offering the following preamble and resolutions:

WHEREAS, There is to be a world's Columbian exposition in Chicago in 1893; and

WHEREAS, In consequence of the fact that the choicest products of the world are to be there displayed, it is expected that citizens in large numbers of all civilized countries will be gathered together there for the purpose of seeing these exhibits; and

WHEREAS, It is to be presumed that many dentists from foreign countries will visit the United States at that time; and

WHEREAS, The time of the exposition will be an opportunity for a great meeting of dentists of the world; and

WHEREAS, It is believed that a great advance in the science and practice of dental and oral surgery would result from a meeting of the dentists of the United States with those from foreign countries who might then be visiting this country; and

WHEREAS, it is desirable that any meeting then held should be at the instance of the American Dental Association and the Southern Dental Association, and organized by a joint committee by them appointed; therefore be it

RESOLVED, That the president of this Association appoints a committee of five, to confer with a like committee appointed by the Southern Dental Association at its last meeting upon this subject, and that this joint committee has power to fill all vacancies, and shall add to its membership either one, three, or five more members as it may deem advisable; and when this committee is so completed it shall be clothed with full power to take such action as it in its judgment may deem best for creating an organization for the purpose of holding a dental meeting in Chicago in 1893, which the reputable dentists throughout the world shall be invited to attend, and that any action that this committee may take in the premises shall be final and binding.

No sooner had this resolution been read than Dr. Allport, of Chicago, sprang to his feet and denounced it as a discourtesy if not a direct insult to the Southern Dental Association, whose committee was then on the floor awaiting an opportunity to submit a similar resolution. While he was in favor of co-operating with the Southern Association in the manner they had proposed, he would not consent to this discourteous method of procedure and moved to lay the resolution on the table to give the committee referred to an opportunity to perform its mission.

A spirited discussion then ensued and motions and counter-motions were made with amazing rapidity and amusing disregard of parliamentary law. The gentlemen participating in the fusilade were Dr. J. H. Thompson, of Topeka, Kas., who moved the adoption of the resolutions;

Dr. Allport, of Chicago, who moved to lay them on the table; Dr. J. A. Swazey, of Chicago, who moved a substitute; Dr. Truman of Baltimore, who moved a reference of the whole subject to a committee of three, with instructions to report; Dr. Stockton, of New Jersey, who wanted the whole matter settled right now; Dr. Brophy, of Chicago, who did not make his desires very clearly understood; Dr. Rhein, of New York, who objected to the plenary powers proposed to be conferred on the committee by the resolutions and therefore favored sending the whole subject to a committee to report; Dr. Patrick, of Belleville, whose interesting speech died in its birth, owing to the sudden and unexpected application of "the previous question," most cruelly moved by a doctor who was evidently anxious to close the debate, and Dr. Cushing, who, after the previous question had been voted, skillfully extricated the convention from the tangle into which it was getting by offering, and getting passed, a resolution to table the whole matter temporarily, so as to afford the committee of the Southern Association an opportunity to present the resolutions adopted by that body, which are, in substance, about the same as those offered by Dr. Gardiner.

Indeed, they are so nearly identical that Dr. Allport in his opposing speech, had denounced the presentation as stealing. The true inwardness of the debate came out afterwards in conversation, when the action of Dr. Gardiner was pronounced to be a sharp move on the part of a Chicago clique to obtain the chairmanship and, perhaps, the lion's share of representation on the proposed committee, to the exclusion of others who ought to have equal rights. The upshot of the affair was that when peace had been restored and some other business had been transacted, Dr. Carpenter, of Atlanta, Ga., chairman of the committee appointed by the Southern Dental Association, was called upon and submitted the resolutions of that body, which were read by the secretary, Dr. Cushing, and unanimously indorsed, except that Dr. Rhein, of New York, still adhered to his objections to the absolute powers to be granted to the committee. The president was thereupon instructed to appoint a committee of five from the American Dental Association, to co-operate with the committee of the Southern Association, which committee will probably be named this evening.

A communication was read from the S. S. White Dental Manufacturing Company, of Philadelphia, which has the contract for publishing the transactions of the association, relating the history of the arrangement under which the publication is made and pointing out the benefits the association derived there-from.

This brought out Dr. Patrick, of Belleville, Ill, who most energetically denounced the Whites and the entire arrangement, objecting to the employment of a firm whose only interest in the association was that which a man would have in the contemplation of a turkey that he was about to devour for his Thanksgiving dinner.

Dr. Crouse, chairman of the executive committee, stated the reasons why he had, when chairman of the finance committe, made the publishing arrangement with the S. S. White Company, whose *Dental Cosmos* is the recognized organ of the dental profession in this country and the authority for the world. He showed how it had been formerly a great expense, one that could not be met without persistent begging, to obtain publication of the transactions of the association, whereas now they were not only saved this expense, but had always a balance of some thousands in the treasury. After some further unimportant debate, resolutions offered by Dr. H. A. Smith, of Cincinnati, indosing the present sytem of publication, were adopted.

The president then read the annual address which reviewed at length the dental laws of the various states, showed that all but five of the states and territories in the Union had such laws, and that nearly all of them differed in essential features from each other. This led to great confusion and inconsistency in the requirements for those practicing dentistry in the different states, and would, if some means were not adopted to rectify the evil, lead to conflict and practical anarchy in the profession. He eloquently urged on the convention the necessity for considering the means for bringing about a conformity in the state dental laws throughout the Union, and made some cogent suggestions in that direction.

The dental laws were divided by the address into four classes—those that demand that both graduates and non-graduates shall be examined by state boards before admission to practice, those recognizing the diplomas of reputable schools and permitting the holders to practice to the exclusion of all others desiring the privilege, those demanding examinations but allowing the favor to graduates only, and those demanding examinations but giving exceptional clauses in favor of the holders of the medical degree. The different kinds of legislation were discussed by President Foster in an able manner. The president favored a unity of action between the state boards and the faculties of the dental colleges, and urged that diplomas which admitted the dentist to practice in one state should be recognized in the other states.

President Foster also urged that congress be petitioned to appoint with suitable rank and pay dentists in the army and navy wherever service of this nature may be required. Another clause recommended to the proper authorities the use of models taken from impressions of the mouth for the identification of criminals. He urged that the models would prove a valuable means of identification and suggested that they would also become a valuable source of study to the ethnologist and physiognomist.

The address contained still another clause recommending the establishment of a headquarters, dental museum and library of the association to be located at Washington.

The president of the association, continuing, said that he had confidence that the government would aid in the work, perhaps furnishing apartments in the Smithsonian Institution. In concluding his address President Foster referred to the thirty years of the association, the work that it has accomplished, the work that it is now doing and will probably do in the future.

At the evening session Dr. Foster, the president, announced the following committee to co-operate with the committee of the Southern Dental Association in preparing for the meeting of the dentists of the world, to be held in Chicago in 1893. Dr. Shepherd, of Boston; Dr. Walker, of New York; Dr. Hunter, of Iowa City; Dr. Noble, of Washington, and Dr. McElhany, of Columbus, Ga. A number of papers were read from section 5 and one by the venerable W. H. Atkinson, of New York, on "Dental Nomenclature."

AUGUST 6TH, WEDNESDAY.

The committee appointed on the first day, on arrangements, for a universal meeting of dentists during the World's Fair at Chicago in 1893, met at 8 A. M. as follows:

From the Southern Dental Association—Dr. J. Y. Crawford, of Nashville, Tenn.; Dr. John Storey, president Southern Dental Association, Dallas, Tex.; Dr. L. D. Carpenter, Atlanta, Ga.; Dr. Barton, Paris, Tex.; Dr. C. E. Stockton, Newark, N. J.; Dr. J. Taft, Cincinnati.

From the American Dental Association—Dr. L. D. Shephard, Boston; Dr. W. W. Walker, New York; Dr. L. D. Noble, Washington, D. C.; Dr. A. O. Hunt, Cincinnati; Dr. Marshall, Chicago; Dr. McElhaney, Augusta, Ga.

Elected at large—Dr. M. W. Foster, Baltimore; Dr. H. J. McKellops, St. Louis, and Dr. H. A. Harlan.

The committee then elected the following permanent officers: President, Dr. W. W. Walker, of New York; secretary, Dr. A. O. Hunt, of Cincinnati; treasurer, Dr.

Marshall, of Chicago. The great importance of this committee can be seen when the following clause, under which it has been created, is read: "And when this committee is so completed it shall be clothed with full power to take such action as it in its Judgment may deem best for creating an organization for the purpose of holding a dental meeting in Chicago in 1893, which the reputable dentists throughout the world shall be invited to attend, and that any action that this committee may take in the premises shall be final and binding."

The Association Proceedings for the second day were as follows: When President Foster had called the meeting to order he introduced the first vice president, Dr. A. W. Harlan, as the presiding officer of the day and work began forthwith. Prior to the meeting, however, expressions of regret were wired on behalf of the association to Dr. W. H. Morgan, of Nashville, Tenn., and Dr. E. T. Darby, of Philadelphia, who are prevented from attending the convention by sickness.

On the call of the sections, Dr. Peirce, chairman of the section on "Dental Education, Literature and Nomenclature," read a long and interesting report. The burden of his argument, as of all or nearly all the arguments being made here, was urging upon the convention the adoption of some means of raising the standard of dental education, and procuring uniformity in the various state laws. Charters were issued too readily to institutions calling themselves dental colleges, but which were established for revenue only, and with little or no regard to real, valuable dental education. He suggested that a committee of five should be appointed to consider and report, at the next annual meeting, upon the advisability of establishing a national board empowered to confer degrees on dentists of exceptional attainments. He animadverted on the comparative slenderness of the attendance, and particularly on the fact that the great bulk of delegates came from but a few states. In support of this he stated that last night, before this morning's arrivals, there were seventy-five delegates from three states, while the other twenty-three only sent about 100 representatives. At the conclusion of the reading the report was handed in in company with papers that had been read in the same section by Dr. W. H. Atkinson, of New York; Dr. A. H. Thompson, of Topeka, Kas.; Dr. C. B. Atkinson, of New York, and Dr. Rodriquez Otto Lengui, of New York.

In compliance with a resolution offered at the close of Dr. Peirce's report, a committee of five, consisting of Dr. A. H. Thompson, of Topeka; Dr. J. Y. Crawford, of Nashville, Tenn.; Dr. C. N. Peirce, of Philadelphia; Dr. H. B. Noble, of Washington, D. C., and Dr. W. W. Walker, of New York, was appointed to consider and report upon those sections of President Foster's address which dealt with the unification of the state dental laws, the proposed employment of dentists in the United States army and navy, and the matter of scholastic education before matriculation of students in the dental colleges. Dr. J. W. Crouse, of Chicago, strenuously opposed the resolution to appoint this committee, on the ground that there had been too many resolutions and too many committees any way, with no appreciative results.

Debate then opened on the report of Dr. Peirce, which was participated in by Dr. H. B. Noble, of Washington, D. C.; Dr. E. Parmly Brown, of New York; Dr. C. S. Stockton, of New Jersey; Dr. A. W. Smith, of Kentucky; Dr. J. Y. Crawford of Tennessee; Dr. J. C. Story, of Dallas, Tex.; Dr. W. W. Allport, of Chicago; Dr. C. L. Goddard, of San Francisco; Dr. P. T. Smith, of Denver, and Dr. James Truman, of Philadelphia. At one time during the progress of the debate considerable heat was engendered by Dr. E. Parmly Brown, of New York, who criticized the dental laws of New Jersey very severely and said that "while he had been a practicing dentist in New York city for thirty-five years he could not practice in New Jersey without being asked a lot of questions by a lot of boys whom he had picked up out of the gutter, given a dental education and set upon their feet."

This brought Dr. W. W. Walker, of New York, to his feet with a blunt contradiction of the picking-up-out-of-the-gutter remark, to which Dr. Brown replied that "for every one of the New Jersey examiners that Dr. Walker could show whom he had not picked up out of the gutter he, Dr. Brown, could show nine whom he had."

At this an unpleasant feeling was apparent till Dr. Stockton, of New Jersey, poured oil on the waters and in a temperate and rather skillful speech, set before the meeting the true meaning of the New Jersey dental laws, which were not such terrible affairs as Dr. E. Parmly Brown had supposed them to be. As the noon recess hour was approaching, Dr. J. W. Crouse, of Chicago, president of the Dental Protective Association, asked for and obtained a suspension of the rules for the purpose of offering a resolution for the appointment of a committee of four, one from each of the grand divisions of the country, to examine and report to the convention on the expenditures and accounts of the Dental Protective Association. This association is a special body organized to fight all bogus patents and defend all suits where its members are parties, and one of the principal matters to be investigated will be the standing and qualifications of the counsel employed for these purposes. The resolution was adopted and the chair appointed as the investigating committee Dr. C. L. Goddard, of San Francisco; Dr. J. Y. Crawford, of Nashville, Tenn.; Dr. W. W. Walker, of New York, and Dr. D. F. H. Gardiner, of Chicago.

Operative dentistry was the subject discussed at length last night and this morning. The report was made by Dr. N. S. Hoff of Michigan, secretary of section 3. The reports and papers presented by Dr. Atkinson, of New

York and Dr. Williams, of Boston, were extremely technical and dealt largely with cases and the best methods of treatment. The discussions were participated in by Dr. M. L. Rhein of New York, Dr. Harlan of Chicago, Dr. Stemmefield of Nashville, Dr. Brophy ot Philadelphia, Dr. Conrad of St. Louis, Dr. Atkinson of New York, Dr. Patrick of Illinois, Dr. Storey of Texas, Dr. P. Browns of New York, Dr. Senaze of Chicago, Dr. Smith of Denver, Dr. Goddard of San Francisco, Dr. Crouse of Chicago, Dr. Crawford of Tennessee and Dr. Truman of Philadelphia.

Section 4 was, after some sharp discussion, passed until evening, to allow the secretary, Dr. W. X. Sudduth, to present a report. Section 4 is devoted to the study of histology and microscopy. Section 5, under the chairmanship of the president, Dr. A. W. Harlan of Chicago, presented a report upon materia medica and therapeutics. This report was, if possible, even more technical, and filled the average layman with more awe than any of the sections preceding it. The paper written by Dr. Harlan deprecated the use of carbolic acid by dentists. The report elicited no discussion, being evidently too deep for even the scientists present, and Chairman H. A. Smith of Cincinnati presented the report for section 6, devoted to the consideration of physiology and etiology. He presented a report upon the tabulation of prehistoric crania, referring to the recent action of the Illinois State Dental Association in starting the investigations of the skulls now in the museums of the country. The best method of labeling teeth was discussed by Dr. Patrick of Illinois who is to have charge of the work of compiling the returns from the museums of the country and Dr. Smith of Denver who has invented a system of dental notation which he claims gives excellent satisfaction. The convention was treated to a paper on systems of dental notation presented by Dr. How of Philadelphia. The discussion resulted in a resolution looking to the unification of the systems of dental notation at the session of the dental congress at Chicago in 1893.

Section 7 was next called upon for its report. The report was presented by Dr. M. L. Rhein of New York. He read a paper on "Alveolal Abscess." The paper was written by Dr. C. Hugenschmidt of Paris, France, and was listened to with interest and attention. Dr. Rhein, a specialist in cases of this nature, then presented a paper on "Alveolal Abscess" which showed great depth of scientific research and was heartily applauded by the convention. After sending a cablegram of congratulations to the International Dental Association now in session in Berlin the convention adjourned until evening.

At the night session the association officers for the ensuing year were chosen as follows: Dr. A. W. Harlan, Chicago, president; Dr. J. D. Patterson, Kansas City, first vice president; Dr. H. B. Noble, Washington, D. C., second vice president; Dr. J. H. Cushing, Chicago, recording secretary; Dr. Fred. A. Levy, Orange, N. J., corresponding secretary; Dr. A. H. Fuller, St. Louis, treasurer.

The time after the election of officers, was devoted to papers illustrated by magic lanters. The paper upon the blood supply of the teeth was presented by Dr. A. O. Hunt of Iowa City, Ia.; the paper upon oral tumors was presented by Dr. W. X. Sudduth of Minneapolis, Minn. At the close of the evening session a full dress ball was given under the direction of Prof. Scard at the parlors of The Elms. The party in honor of the dentists was greatly appreciated and enjoyed by all.

The first lady elected member of the American Dental Association was admitted to day. The lady is Mrs. Emma Eames Chase, daughter of the distinguished doctor and dentist, W. Eames of St. Louis, Mo., and wife of Mr. Harry Chase, the distinguished marine painter, recently deceased. Mrs. Chase is practicing with her father in an office in St. Louis and has acquired considerable reputation as a dentist, writer and artist.

At the meeting of the association last night the question of the place of meeting was left in the hands of the committee of arrangements.

AUGUST 7TH, THURSDAY.

Vice President Harlan presided at the session this morning. Dr. C. N. Pierce, chairman of section No. 2, presented the report upon dental education. The present number of colleges teaching dentistry in the United States was stated to be thirty-three. From these schools last year 963 persons graduated. The total number of graduates from 1886 to 1890 was fixed at 3,605. Dr. Pierce compared the rapid growth in the number of dental graduates to the remarkable increase in the number of graduates of medical schools, an increase that has made the medical profession of the United States the wonder and surprise of the world. He thought the three years' course to be a step in the right direction and insisted that state boards to examine the graduates are an absolute necessity, in view of the present condition of things.

A resolution favoring the unification of the state laws in regard to dental practitioners and asking for the appointment of a committee of five, to report at the next meeting, carried unanimously.

Telegrams of condolence were sent to Dr. W. H. Morgan of Nashville, Tenn., and Dr. E. T. Darby of Philadelphia. Both of the gentlemen were former presidents of the association and both are seriously ill at the present time.

The discussion of the papers presented by section 2, upon the subject of dental education, was lengthy and not always to the point. Perhaps the best address was that made by Dr. Truman of Philadelphia. He held that the present stage of dental education is largely transitional, the passing from a period without law to a period when the profession and the people are to be injured by the law. A quarter of a century's work as an educator has convinced Dr. Truman that there are radical defects in the present methods of teaching. He favored more of the practical, less of the didactic, more of demonstrations, less of lectures. He thought the true method in the government of dental colleges would be to aim for liberality of action as well as unity of action. He thought a little more encouragement, a little less criticism for the dental schools of the country would be a benefit in many ways.

Dr. Noble of Washington, D. C., told of the methods employed and the work accomplished for years in trying to get congress to pass a dental law for the District of Columbia and the territories. The committee had labored in the face of the greatest discouragements, sometimes getting the measure through one house of congress, sometimes through the other. He had been assured by Senator McMillan of Michigan that the bill would undoubtedly pass this year. Dr. Brown of New York objected to the different laws in the different states. He objected to the laws of New Jersey because they require dentists, no matter how high their standing, to undergo an examination. He thought it hardly right that a man of years of practice and a reputation extending all over the country should be placed upon the level with recent graduates from the different schools. Dr. Stockton came to the rescue of the New Jersey law. While it might be a little severe in the letter it would prove all right in the spirit and the application, since no board of examiners would be likely to refuse men of Dr. Brown's standing the right to practice. He thought that the law would work well in the long run and insisted that the New Jersey association be ranked among the very first in the land.

Dr. A. W. Smith of Kentucky favored simplicity in legislation. He thought that the line of demarcation ought to be drawn between the true dentist and the poor one without a long preamble. Dr. Crawford of Tennessee in an eloquent speech argued in favor of simplicity, generosity and justice in matters of legislation. The violation of the code of ethics should be punished. As for having too many dentists worthy of the name in the country, Dr. Crawford thought it not possible. Dr. Story of Texas, in a graphic and humorous address, pointed out the dangers and difficulties witnessed in his state in securing and enforcing dental laws. Time should not be a requisite for granting a diploma. Knowledge should be the only requisite. The standard should be placed high, but a diploma should be a passport to the profession and a man holding a diploma should be allowed to practice. Dr. Alport believed that diplomas should be passports, but unfortunately they were not because of the low order of some of the schools. Dr. Goddard explained the workings of the California laws governing schools and graduates. Dr. Smith favored moral suasion and the elevation of the profession by study and hard work.

Dr. J. W. Crouse of Chicago succeeded in obtaining the floor for a few minutes. He made an earnest plea for the dental protective association, urging that it had saved members of the profession half a million dollars by preventing manufacturing establishments invented by members of the profession from obtaining patents on inventions designed by the inventors for the common good of all. After listening to the address the convention adjourned for dinner. The afternoon was spent in committee meetings. At the evening session the subject of dental education, as reported in section No. 2, was again taken up by the convention.

AUGUST 8TH., FRIDAY.

There was merely nothing performed this morning. Most of the dentists were scattered over the grounds or enjoying themselves on the veranda of The Elms hotel. One and all speak in the very highest terms of Excelsior Springs and the splendid accommodations and courteous treatment they have experienced. Last night at an impromtu jollification given by the newly elected president, Dr. Harlan, after the close of the dress ball given in their honor in The Elms dining room, Dr. Truman, of Philadelphia, voiced the sentiments of the entire body. He said, in response to the toast proposed by Dr. W. W. Walker of New York, that last year, when the convention met at Saratoga and they had met with several disappointments, the cry had been excelsior. They looked to the future to bring the better things, and they had found better things, at Excelsior Springs. Many of them had thought that in coming into a part of the country which many Eastern people still consider wild and lawless they would have to put up with many discomforts. Instead of this, they had found greater comfort, better hotel accommodations and more liberal and courteous treatment than at any place they had met in all the years of their organization and existence as an association. There was no exception to this broad assertion that could be sustained, and, for his part he should depart with the liveliest feelings of satisfaction with The Elms, the springs, the lovely scenery and the kindly treatment extended to them by the managers of the Excelsior Springs Company.

Other speeches followed, all in the same strain of eulogy, and before the convention adjourned, by way of giving official expression to the feeling, the following preamble and resolutions were entered of record on the secretary's books:

"During the first week in August the American Dental Association, the National Association of Dental Examiners, the National Association of Dental Faculties and the Dental Protective Association, associated with whom were many members of the Southern Dental Association, by way of expressing their several and collective opinions of the admirable treatment they all received at the hands of the Excelsion Springs Company's managers, and their appreciation of the excellence of The Elms hotel, have caused the following resolution to be offered at a meeting of theAmerican Dental Association and the same was passed unanimously:

"RESOLVED, That in selecting Excelsior Springs, Mo., as the place for holding our thirteenth annual meeting this association has been particularly fortunate. Whether we regard the beauties of the surroundings, the variety of health giving waters, the excellence of the hotel or the courteous, liberal and accommodating spirit of the company's manager's, we can especially recommend Excelsior Springs, Mo., as a desirable place for holding literary and scientific conventions and as possessing unusual advantages as a health and pleasure resort."

